# The first real-world evidence on dose-dense methotrexate, vinblastine, doxorubicin, and cisplatin followed by switch maintenance avelumab in advanced urothelial carcinoma: a propensity score-matched study

**DOI:** 10.1007/s10147-025-02729-x

**Published:** 2025-03-03

**Authors:** Satoru Taguchi, Taketo Kawai, Yoshiaki Kurokawa, Naoki Saegusa, Masahiro Yamamoto, Yoshiki Ambe, Kazuki Honda, Kazuki Maki, Yoichi Fujii, Jimpei Miyakawa, Yuumi Tokura, Hazuki Inoue, Tomoyuki Kaneko, Takehiro Tanaka, Katsuhiko Nara, Jun Kamei, Shigenori Kakutani, Yuta Yamada, Aya Niimi, Daisuke Yamada, Tappei Takada, Tohru Nakagawa, Haruki Kume

**Affiliations:** 1 Department of Urology, Graduate School of Medicine, The University of Tokyo, 7-3-1 Hongo, Bunkyo-ku, Tokyo 113-8655 Japan; 2 Department of Urology, International University of Health and Welfare Ichikawa Hospital, 6-1-14 Konodai, Ichikawa, Chiba 272-0827 Japan; 3 Department of Urology, Teikyo University School of Medicine, 2-11-1 Kaga, Itabashi-ku, Tokyo 173-8605 Japan; 4 Department of Pharmacy, The University of Tokyo Hospital, 7-3-1 Hongo, Bunkyo-ku, Tokyo 113-8655 Japan

**Keywords:** Avelumab, Dose-dense MVAC, Enfortumab vedotin, Pembrolizumab, Urothelial carcinoma

## Abstract

**Background:**

Dose-dense methotrexate, vinblastine, doxorubicin, and cisplatin (dd-MVAC) is an established regimen for advanced urothelial carcinoma (aUC). Although platinum-based chemotherapy, typically gemcitabine and cisplatin, followed by switch maintenance avelumab has been a recommended strategy for aUC, no study has evaluated outcomes of dd-MVAC followed by avelumab therapy.

**Methods:**

We reviewed 71 patients treated with first-line dd-MVAC for aUC at two university hospitals between 2018 and 2024. Overall survival (OS) and progression-free survival (PFS) were assessed as endpoints. Additionally, among patients who achieved ≥ stable disease, we performed propensity score matching between patients with and without avelumab to balance their background characteristics.

**Results:**

Of 71 patients, 49 (69%) experienced disease progression and 30 (42%) died during the median follow-up of 13 months. Median OS and PFS were 24 and 7 months, respectively. Among 59 patients who achieved ≥ stable disease after completion of dd-MVAC, 35 received switch maintenance avelumab, while the remaining 24 did not. After propensity score matching, patients with avelumab had significantly longer OS and PFS (both: not reached) than those without (OS: 28 months; PFS: 7 months).

**Conclusions:**

We herein report outcomes of dd-MVAC followed by switch maintenance avelumab in real-world patients with aUC for the first time. Avelumab therapy was significantly associated with longer survival in patients who achieved ≥ stable disease after first-line dd-MVAC. Given the excellent survival outcomes, dd-MVAC followed by switch maintenance avelumab may still be a valid option for aUC even in the new treatment paradigm as typified by enfortumab vedotin and pembrolizumab.

**Supplementary Information:**

The online version contains supplementary material available at 10.1007/s10147-025-02729-x.

## Introduction

Platinum-based chemotherapy has been the mainstay of therapy for advanced urothelial carcinoma (aUC) since the 1980s [1, 2]. Representative regimens include gemcitabine and cisplatin (GC) [3], methotrexate, vinblastine, doxorubicin, and cisplatin (MVAC) [4], and dose-dense MVAC (dd-MVAC) [5–9]. Compared to standard MVAC (4-week cycle), dd-MVAC (2-week cycle) was designed to enhance dose intensities of doxorubicin and cisplatin with the aid of prophylactic granulocyte colony-stimulating factor administration [5, 6]. Although the first report on dd-MVAC for aUC did not show its survival superiority over standard MVAC [5], a subsequent report (seven-year update) successfully demonstrated a survival advantage for dd-MVAC [6]. Accordingly, dd-MVAC has been recommended as an efficacious first-line regimen for aUC [10, 11], albeit its relatively low utilization rate in the real-world setting. More recently, the VESPER trial revealed that dd-MVAC provided better local control, overall survival (OS), and progression-free survival (PFS) than GC in the perioperative (especially, neoadjuvant) use for muscle-invasive bladder cancer [7–9], which has led to a recommendation for dd-MVAC as a preferred regimen in the perioperative setting [10, 11].

On the other hand, switch maintenance avelumab was introduced in 2020, based on the JAVELIN Bladder 100 trial which showed that the treatment significantly improved OS in patients with aUC who had not progressed with first-line chemotherapy (four to six cycles of GC or gemcitabine and carboplatin [GCa]) [12, 13]. As such, switch maintenance avelumab following first-line platinum-based chemotherapy has become a recommended strategy for aUC [10, 11]. In this “JAVELIN paradigm”, several studies have reported real-world evidence on switch maintenance avelumab in aUC [14–16]. However, these studies mostly used GC or GCa as first-line chemotherapy but not dd-MVAC, and evidence on dd-MVAC followed by avelumab therapy is totally lacking. Therefore, this study aimed to assess the outcomes of dd-MVAC followed by switch maintenance avelumab in real-world patients with aUC for the first time.

## Materials and methods

### Patients and treatments

We reviewed 71 consecutive patients who were treated with first-line dd-MVAC for aUC at two university hospitals between August 2018 and March 2024. (For reference, during the same period, 20 and 39 patients received first-line GC and GCa, respectively.) This retrospective multicenter study was approved by the Institutional Review Board of the Graduate School of Medicine and Faculty of Medicine, The University of Tokyo (approval number: 2024246NI), as well as that of Teikyo University School of Medicine. An opt-out approach was used to obtain informed consent from patients.

**Fig. 1:**
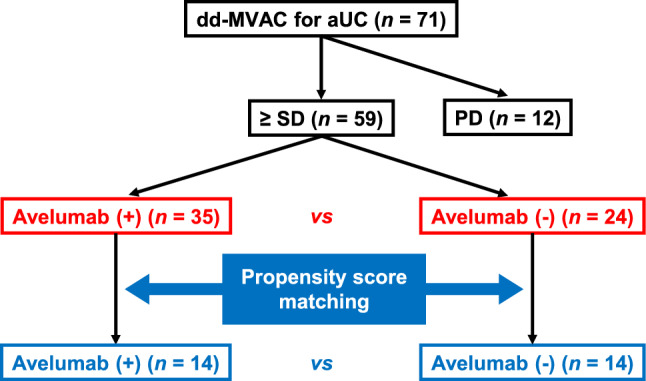
Flowchart presenting the study selection process. *aUC* advanced urothelial carcinoma; *dd-MVAC* dose-dense methotrexate, vinblastine, doxorubicin, and cisplatin; *PD* progressive disease; *SD* stable disease

Of 71 patients, 59 achieved ≥ stable disease (SD) after completion of first-line dd-MVAC, whereas the remaining 12 developed progressive disease (PD). Of the 59 patients with ≥ SD, 35 received switch maintenance avelumab, while the remaining 24 did not. We performed propensity score matching (PSM) between patients with and without avelumab to balance background characteristics of the two groups. A flowchart presenting the study selection process is shown in Fig. 1.

The detailed schedule of dd-MVAC is described elsewhere [17]. Briefly, the full-dose regimen comprised methotrexate 30 mg/m^2^ on day 1, vinblastine 3 mg/m^2^ on day 2, doxorubicin (or pirarubicin) 30 mg/m^2^ on day 2, cisplatin 70 mg/m^2^ on day 2, and pegfilgrastim 3.6 mg/body on day 4, administered every 2 weeks for a total of six cycles. However, given the high frequency of severe adverse events necessitating the early termination of treatment after the first cycle of full dose administration, we adopted a dose reduction protocol wherein the first cycle of methotrexate and cisplatin was administered at 75% of the full dose and only patients who did not experience significant adverse events in the first cycle then proceeded with the full dose from the second cycle onward. Additional dose reductions were also considered based on the Cockcroft-Gault creatinine clearance and severe adverse events during the course of chemotherapy. Treatment response was assessed by computed tomography according to the Response Evaluation Criteria in Solid Tumours (RECIST) version 1.1. at the end of the 3rd and 6th cycles in all cases. Chemotherapy was discontinued if disease progression was confirmed or if the patient's general condition deteriorated due to adverse events. It should be noted that patients who developed PD after the first three cycles of dd-MVAC discontinued the therapy, while those who achieved ≥ SD proceeded to the next three cycles [17]. Switch maintenance avelumab was intravenously administered at a dose of 10 mg/kg every 2 weeks to some patients who achieved ≥ SD after completion of first-line dd-MVAC [12, 13].

### Endpoints and follow-up

We assessed the associations of clinicopathological variables with OS and PFS. OS was defined as the time from the initiation of dd-MVAC to death. PFS was defined as the time from the initiation of dd-MVAC to disease progression according to RECIST version 1.1 or death, whichever occurred first. Treatment responses, including complete response, partial response, SD, and PD, were also assessed according to RECIST version 1.1. All patients underwent evaluations every 1–3 months that included routine blood tests and computed tomography. Treatment discontinuation and/or regimen change were considered at the attendant physician’s discretion if disease progression was confirmed or if the patient's general condition deteriorated due to adverse events. The most commonly used later-line regimens after dd-MVAC with and without avelumab were enfortumab vedotin (EV) and pembrolizumab, respectively. The patients’ charts were comprehensively reviewed, and the status of each patient was assessed through office visits and/or telephone calls. Follow-up information was obtained as of November 2024.

### Statistical analysis

For PSM, multivariable logistic regression analysis was used to calculate propensity scores, and matching was conducted on the logit of the propensity score using nearest neighbor matching with a caliper of 0.20. Before and after PSM, the significance of the differences of clinicopathological variables between patients with and without avelumab were evaluated using Student’s *t*-test for continuous variables and the χ^2^ test for categorical variables.

Before and after PSM, OS and PFS were estimated using the Kaplan–Meier method and compared using the log-rank test. Univariable and multivariable Cox proportional hazard regression analyses for OS and PFS were conducted before PSM. All statistical analyses were performed using JMP Pro version 17 (SAS Institute, Cary, NC, USA). *P* < 0.05 was considered significant.

## Results

### Analyses of all patients treated with dd-MVAC

Table 1 presents characteristics of all patients treated with dd-MVAC (*n* = 71). After completion of dd-MVAC, a total of 59 (83%) patients achieved ≥ SD, and 35 (49%) received subsequent switch maintenance avelumab. Median cycles of avelumab therapy were 10 (IQR: 6–22) cycles. Furthermore, the median treatment-free interval from the end of dd-MVAC to the start of avelumab was 14 (interquartile range [IQR]: 4–30) days, or 2 (IQR: 0–4) weeks. The treatment-free interval was < 4 weeks in 23 (66%) patients, 4–10 weeks in 8 (23%), and > 10 weeks in 4 (11%).

**Table 1:** Characteristics of all patients treated with dd-MVAC (*n* = 71)

Parameter	Value
Age, years, median (IQR)	72 (62–79)
Sex, no. (%)	
Male	48 (68)
Female	23 (32)
ECOG PS, no. (%)	
0	54 (76)
1	14 (20)
≥ 2	3 (4)
Primary site, no. (%)	
Bladder only	38 (54)
Upper urinary tract or both	33 (46)
Resection of primary site, no. (%)	15 (21)
Cockcroft-Gault creatinine clearance, mL/min, median (IQR)	56.1 (43.8–71.7)
Prior perioperative chemotherapy, no. (%)	9 (13)
Cycles of dd-MVAC, cycles, median (IQR)	6 (3–6)
Response to dd-MVAC, no. (%)	
CR	7 (10)
PR	36 (51)
SD	16 (23)
PD	12 (17)
Subsequent switch maintenance avelumab, no. (%)	35 (49)
Lymph node metastasis, no. (%)	42 (59)
Visceral metastasis, no. (%)	36 (51)
Lung metastasis, no. (%)	23 (32)
Bone metastasis, no. (%)	14 (20)
Liver metastasis, no. (%)	11 (15)

*CR* complete response; *dd-MVAC* dose-dense methotrexate, vinblastine, doxorubicin, and cisplatin; *ECOG PS* Eastern Cooperative Oncology Group performance status; *IQR* interquartile range; *PD* progressive disease; *PR* partial response; *SD* stable disease

**Fig. 2:**
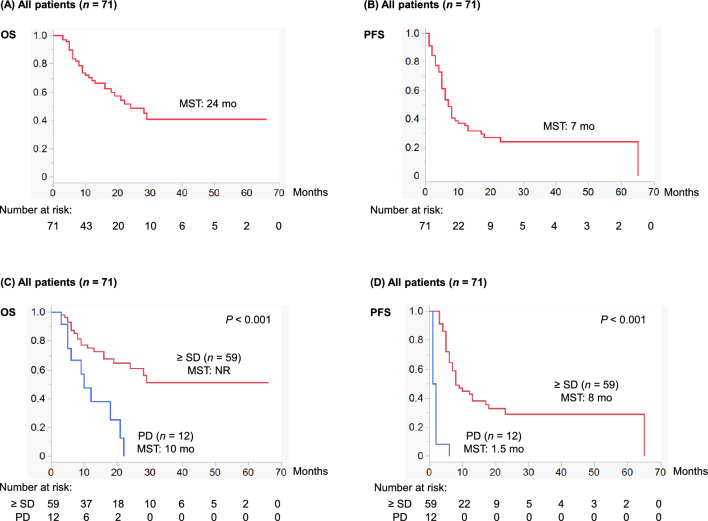
The upper half presents Kaplan–Meier curves depicting (**A**) OS and (**B**) PFS in all patients treated with dd-MVAC (*n* = 71). The lower half presents Kaplan–Meier curves depicting (**C**) OS and (**D**) PFS according to the response to dd-MVAC (≥ SD vs. PD) in all patients (*n* = 71). *dd-MVAC* dose-dense methotrexate, vinblastine, doxorubicin, and cisplatin; *MST* median survival time; *NR* not reached; *OS* overall survival; *PD* progressive disease; *PFS* progression-free survival; *SD* stable disease

Of 71 patients, 49 (69%) experienced disease progression and 30 (42%) died during the median follow-up of 13 (IQR: 6–24) months. All the 30 fatal cases died of aUC and none died from other causes. Figure 2 shows Kaplan–Meier curves depicting OS and PFS in all the patients (*n* = 71). Median OS and PFS were 24 (IQR: 9–not reached [NR]) and 7 (IQR: 4–23) months, respectively (Figs. 2A and B). Furthermore, based on treatment response at the completion of dd-MVAC, median OS and PFS in patients with ≥ SD were NR (IQR: 13–NR) and 8 (IQR: 5–65) months, respectively, whereas those in patients with PD were 10 (IQR: 5.5–21) and 1.5 (IQR: 1–2) months, respectively (Figs. 2C and D).

Univariable and multivariable Cox proportional hazard regression analyses for OS and PFS in all patients (*n* = 71) are shown in Supplementary Tables 1 and 2, respectively. All variables related to first-line dd-MVAC were included in the analyses, except for subsequent switch maintenance avelumab (a post-treatment factor). Multivariable analyses identified female sex, Eastern Cooperative Oncology Group performance status ≥ 2 (vs. ≤ 1), and liver metastasis as independent poor prognostic factors for OS (Supplementary Table 1). Meanwhile, PD after completion of dd-MVAC (vs. ≥ SD) and liver metastasis were independent poor prognostic factors for PFS (Supplementary Table 2).

### Assessing the survival-prolonging effect of switch maintenance avelumab using PSM

To assess the survival-prolonging effect of switch maintenance avelumab after first-line dd-MVAC, we compared outcomes between patients with and without avelumab among those achieving ≥ SD after completion of dd-MVAC.

Table 2 presents characteristics of patients with ≥ SD before and after PSM. Before PSM (*n* = 59), there was a significant difference in cycles of dd-MVAC between patients with and without avelumab, as well as marginal (*P* ≥ 0.05) differences in primary site and lung metastasis. After PSM (*n* = 28), characteristics between patients with and without avelumab became more balanced, with no significant parametric differences.

**Table 2:** Characteristics of patients with ≥ SD before and after PSM

	Before PSM (*n* = 59)			After PSM (*n* = 28)		
Parameter	With avelumab (*n* = 35)	Without avelumab (*n* = 24)	*P*	With avelumab (*n* = 14)	Without avelumab (*n* = 14)	*P*
Age, years, median (IQR)	72 (63–77)	72 (65–80)	0.55^a^	73 (64–80)	72 (65–79)	0.78^a^
Sex, no. (%)			0.59^b^			1.00^b^
Male	24 (69)	18 (75)		11 (79)	11 (79)	
Female	11 (31)	6 (25)		3 (21)	3 (21)	
ECOG PS, no. (%)			0.40^b^			1.00^b^
≤ 1	34 (97)	24 (100)		14 (100)	14 (100)	
≥ 2	1 (3)	0 (0)		0 (0)	0 (0)	
Primary site, no. (%)			0.072^b^			0.45^b^
Bladder only	15 (43)	16 (67)		6 (43)	8 (57)	
Upper urinary tract or both	20 (57)	8 (33)		8 (57)	6 (43)	
Resection of primary site, no. (%)	8 (23)	5 (21)	0.85^b^	4 (29)	3 (21)	0.66^b^
Cockcroft-Gault creatinine clearance, mL/min, median (IQR)	55.0 (42.6–67.5)	54.3 (41.6–71.2)	0.66^a^	53.4 (42.4–73.2)	58.2 (43.1–73.1)	0.68^a^
Prior perioperative chemotherapy, no. (%)	6 (17)	3 (13)	0.63^b^	2 (14)	2 (14)	1.00^b^
Cycles of dd-MVAC, cycles, median (IQR)	6 (6–6)	5.5 (3–6)	0.007^a^	6 (6–6)	6 (5–6)	0.75^a^
Response to dd-MVAC, no. (%)			0.37^b^			0.66^b^
≥ PR	27 (77)	16 (67)		11 (79)	10 (71)	
SD	8 (23)	8 (33)		3 (21)	4 (29)	
Lymph node metastasis, no. (%)	22 (63)	12 (50)	0.33^b^	7 (50)	9 (64)	0.45^b^
Visceral metastasis, no. (%)	14 (40)	12 (50)	0.45^b^	7 (50)	5 (36)	0.45^b^
Lung metastasis, no. (%)	7 (20)	10 (42)	0.071^b^	6 (43)	4 (29)	0.43^b^
Bone metastasis, no. (%)	5 (14)	3 (13)	0.84^b^	1 (7)	1 (7)	1.00^b^
Liver metastasis, no. (%)	4 (11)	4 (17)	0.56^b^	1 (7)	2 (14)	0.54^b^

*dd-MVAC* dose-dense methotrexate, vinblastine, doxorubicin, and cisplatin; *ECOG PS* Eastern Cooperative Oncology Group performance status; *IQR* interquartile range; *PR* partial response; *PSM* propensity score matching; *SD* stable disease

^a^Student’s *t*-test

^b^χ^2^ test

**Fig. 3:**
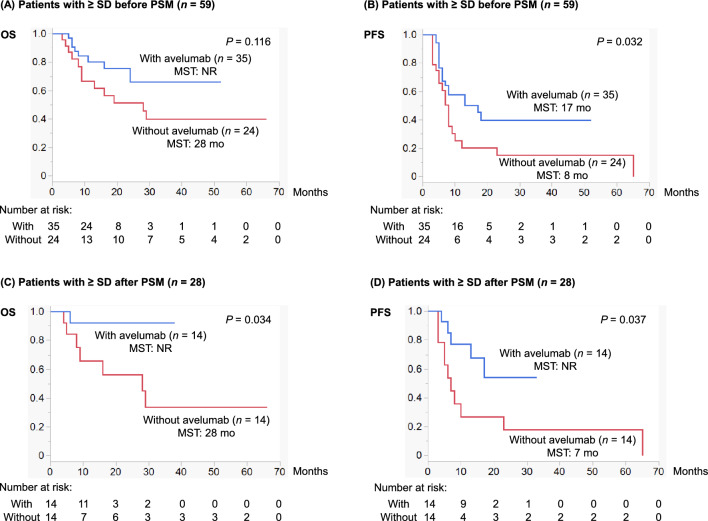
The upper half presents Kaplan–Meier curves depicting (**A**) OS and (**B**) PFS according to the implementation of switch maintenance avelumab (with vs. without) in patients with ≥ SD before PSM (*n* = 59). The lower half presents Kaplan–Meier curves depicting (**C**) OS and (**D**) PFS according to the implementation of switch maintenance avelumab (with vs. without) in patients with ≥ SD after PSM (*n* = 28). *MST* median survival time; *NR* not reached; *OS* overall survival; *PFS* progression-free survival; *PSM* propensity score matching; *SD* stable disease

Figure 3 shows Kaplan–Meier curves depicting OS and PFS according to the implementation of switch maintenance avelumab in patients with ≥ SD. Before PSM (*n* = 59), median OS and PFS in patients with avelumab were NR (IQR: 24–NR) and 17 (IQR: 6–NR) months, respectively, whereas those in patients without avelumab were 28 (IQR: 9–NR) and 8 (IQR: 4–12) months, respectively. There was a significant difference in PFS (*P* = 0.032) but not in OS (*P* = 0.116; only a non-significant trend) between patients with and without avelumab (Figs. 3A and B). After PSM (*n* = 28), median OS and PFS in patients with avelumab were NR (IQR: NR–NR) and NR (IQR: 13–NR) months, respectively, whereas those in patients without avelumab were 28 (IQR: 9–NR) and 7 (IQR: 5–23) months, respectively. There were significant differences both in OS (*P* = 0.034) and PFS (*P* = 0.037) between patients with and without avelumab (Figs. 3C and D).

For reference, subsequent treatments after dd-MVAC with avelumab (*n* = 35) consisted of EV (*n* = 11), pembrolizumab (*n* = 2), dd-MVAC (*n* = 1), GC (*n* = 1), and none (*n* = 20). Meanwhile, those after dd-MVAC without avelumab (*n* = 36; including 12 cases with PD) comprised pembrolizumab (*n* = 18), GCa (*n* = 2), cystectomy (*n* = 2), nephroureterectomy (*n* = 1), metastasectomy (*n* = 1), and none (*n* = 12).

## Discussion

This study was the first to assess outcomes of dd-MVAC followed by switch maintenance avelumab in real-world patients with aUC. Firstly, it reported outcomes in all patients treated with dd-MVAC (*n* = 71), as well as assessed prognostic factors associated with OS and PFS. Median OS and PFS were 24 and 7 months, respectively, and liver metastasis was identified as an independent poor prognostic factor for both OS and PFS, the latter of which was consistent with previous studies that reported liver metastasis as a strong poor prognosticator for aUC [18, 19]. Secondly, this study compared outcomes between patients with and without avelumab therapy among patients with ≥ SD after first-line dd-MVAC (*n* = 59). Finally, it compared outcomes between the two groups using PSM to balance their background characteristics. The differences in PFS between the groups were significant both before and after PSM, while a marginal difference in OS before PSM changed into a significant difference after PSM, presumably because potential confounders which had narrowed the difference were eventually adjusted by PSM, or merely because of variation in computational results due to the small sample size. Nevertheless, the study concluded that switch maintenance avelumab significantly prolonged both OS and PFS following first-line dd-MVAC in real-world patients with aUC even after PSM.

As already described in Introduction, dd-MVAC, an enhanced version of MVAC, has been demonstrated to have a survival advantage over standard MVAC in the salvage setting [5, 6], as well as to have better local control and survival than GC in the perioperative setting [7–9]. Therefore, dd-MVAC has been recommended as a preferred regimen in both salvage and perioperative settings [10, 11]. Nevertheless, dd-MVAC seems to be less frequently used in real-world clinical practice than other common regimens such as GC, presumably due to its high toxicity (and thereby difficulty in its management). As such, real-world evidence on dd-MVAC has been generally lacking especially in the salvage setting.

Meanwhile, switch maintenance avelumab has been shown to have a survival benefit in patients with aUC who did not have disease progression with first-line chemotherapy (GC or GCa) in the JAVELIN Bladder 100 trial [12]. Updated results of the trial after ≥ 2 years of follow-up revealed that median OS was 23.8 months in the avelumab group as compared with 15.0 months in the control group (*P* = 0.0036) with 19.5% of patients receiving ≥ 2 years of avelumab therapy [13]. Switch maintenance avelumab following platinum-based chemotherapy has therefore become recommended strategy for aUC [10, 11], and several studies have reported its real-world outcomes [14–16]. A French multicenter study, termed AVENANCE, collected 595 patients with aUC undergoing avelumab from 82 institutions. It reported that median OS and PFS from avelumab initiation were 21.3 and 5.7 months, respectively, and that common first-line regimens included GCa (61%), GC (28%), and dd-MVAC (4%) [14]. A Japanese multicenter study, termed J-AVENUE, analyzed 79 patients from 15 institutions, and reported that median PFS from avelumab initiation was 6.1 months. Its common first-line regimens were GC (63%) and GCa (30%), whereas only two (3%) patients received dd-MVAC. It also reported that the median treatment-free interval from the end of chemotherapy to the start of avelumab was 4.9 (IQR: 3.1–8.0) weeks, which seemed longer than the present study [15]. Another Japanese study included 27 patients treated with avelumab, and first-line regimens mostly comprised GC and GCa (both: 44%) while only one (4%) patient underwent dd-MVAC [16]. As seen above, previous studies generally used GC or GCa as first-line chemotherapy but not dd-MVAC, and therefore, evidence on dd-MVAC followed by switch maintenance avelumab is totally lacking. In this regard, the present study will add distinctive evidence to the field.

Recently, the treatment strategy for aUC has been drastically changed with the emergence of new first-line regimens such as EV and pembrolizumab [20] and GC and nivolumab [21]. In this new paradigm, conventional platinum-based chemotherapy (typically, GC or GCa) followed by switch maintenance avelumab will be less frequently used in the first-line setting. However, compared to the outcomes of the EV-302 trial assessing first-line EV and pembrolizumab (OS: 31.5 months; PFS: 12.5 months) [20], the present results of dd-MVAC followed by avelumab therapy looks satisfactory (OS: NR; PFS: 17 month–NR), while with the caveat that the avelumab cohort did not include patients with PD by its nature. Therefore, first-line dd-MVAC followed by switch maintenance avelumab may still be a valid option in the new treatment paradigm. Furthermore, platinum-based chemotherapy (GC, GCa, or dd-MVAC) followed by avelumab therapy will be used as later-line treatment after failure of first-line EV and pembrolizumab [22].

The limitations of this study included its retrospective design and small sample size. This study did not collect data on the toxicity profile and relative dose intensity of dd-MVAC. However, our recently published article reported the detailed toxicity profile, relative dose intensity, dose reduction protocol, and completion rate of dd-MVAC at our institutions [17]. Survival analyses of avelumab therapy according to the response to dd-MVAC, cycles of dd-MVAC, or the treatment-free interval could not be fully justified due to the small sample size of this subgroup (*n* = 35). In addition, the frequent use (11 of 35, 31%) of EV as subsequent therapy after avelumab might prolong survival in the avelumab group. Nevertheless, this is the first study reporting on real-world outcomes of dd-MVAC followed by switch maintenance avelumab in aUC.

In conclusion, avelumab therapy was significantly associated with longer survival in patients who achieved ≥ SD after first-line ddMVAC even after PSM. Given the excellent survival outcomes of the strategy, dd-MVAC followed by switch maintenance avelumab may still be a valid option for aUC even in the new treatment paradigm as typified by EV and pembrolizumab.

## Supplementary Information

Below is the link to the electronic supplementary material.

Supplementary file1 (DOCX 25 KB)

Supplementary file2 (DOCX 25 KB)

## Data Availability

The datasets generated during and/or analyzed during the current study are available from the corresponding author upon reasonable request.

## Abbreviations

aUC: Advanced urothelial carcinoma
dd-MVAC: Dose-dense methotrexate, vinblastine, doxorubicin, and cisplatin
EV: Enfortumab vedotin
GC: Gemcitabine and cisplatin
GCa: Gemcitabine and carboplatin
IQR: Interquartile range
MVAC: Methotrexate, vinblastine, doxorubicin, and cisplatin
NR: Not reached
OS: Overall survival
PD: Progressive disease
PFS: Progression-free survival
PSM: Propensity score matching
RECIST: Response Evaluation Criteria in Solid Tumours
SD: Stable disease
