# Supplementary material for: The first real-world evidence on dose-dense methotrexate, vinblastine, doxorubicin, and cisplatin followed by switch maintenance avelumab in advanced urothelial carcinoma: a propensity score-matched study
Source: Int J Clin Oncol. 2025 Mar 3;30(5):984–92. doi: 10.1007/s10147-025-02729-x (PMC12014834; doi:10.1007/s10147-025-02729-x)
Supplement: Supplementary file 1 — Supplementary file1 (DOCX 25 KB) [file 10147_2025_2729_MOESM1_ESM.docx]

**Supplementary Table 1** Univariable and multivariable Cox proportional hazard regression analyses of OS in all patients (*n* = 71).

| Parameter | Univariable | | Multivariable | |
| --- | --- | --- | --- | --- |
|  | HR (95% CI) | *P* | HR (95% CI) | *P* |
| Age (continuous) | 0.98 (0.94 to 1.01) per score | 0.15 |  |  |
| Sex (female vs. male) | 4.28 (2.00 to 9.15) | < 0.001 | 4.32 (1.97 to 9.51) | < 0.001 |
| ECOG PS (≥ 2 vs. ≤ 1) | 7.95 (1.70 to 37.30) | 0.009 | 4.75 (0.89 to 25.48) | 0.069 |
| Primary site (upper urinary tract or both vs. bladder only) | 1.20 (0.58 to 2.48) | 0.62 |  |  |
| Resection of primary site (yes vs. no) | 1.04 (0.45 to 2.43) | 0.93 |  |  |
| Cockcroft-Gault creatinine clearance (continuous) | 1.00 (0.98 to 1.02) per score | 0.94 |  |  |
| Prior perioperative chemotherapy (yes vs. no) | 1.35 (0.51 to 3.55) | 0.54 |  |  |
| Cycles of dd-MVAC (continuous) | 0.84 (0.71 to 1.01) per score | 0.069 |  |  |
| Response to dd-MVAC (≥ SD vs. PD) | 0.27 (0.12 to 0.60) | 0.001 | 0.52 (0.22 to 1.21) | 0.13 |
| Lymph node metastasis (yes vs. no) | 1.65 (0.75 to 3.60) | 0.21 |  |  |
| Lung metastasis (yes vs. no) | 1.97 (0.96 to 4.06) | 0.064 |  |  |
| Bone metastasis (yes vs. no) | 1.81 (0.83 to 3.97) | 0.14 |  |  |
| Liver metastasis (yes vs. no) | 3.37 (1.45 to 7.83) | 0.005 | 3.82 (1.57 to 9.26) | 0.003 |

CI, confidence interval; dd-MVAC, dose-dense methotrexate, vinblastine, doxorubicin, and cisplatin; ECOG PS, Eastern Cooperative Oncology Group performance status; HR, hazard ratio; OS, overall survival; PD, progressive disease; SD, stable disease
