# Supplementary material for: The first real-world evidence on dose-dense methotrexate, vinblastine, doxorubicin, and cisplatin followed by switch maintenance avelumab in advanced urothelial carcinoma: a propensity score-matched study
Source: Int J Clin Oncol. 2025 Mar 3;30(5):984–92. doi: 10.1007/s10147-025-02729-x (PMC12014834; doi:10.1007/s10147-025-02729-x)
Supplement: Supplementary file 2 — Supplementary file2 (DOCX 25 KB) [file 10147_2025_2729_MOESM2_ESM.docx]

**Supplementary Table 2** Univariable and multivariable Cox proportional hazard regression analyses of PFS in all patients (*n* = 71).

| Parameter | Univariable | | Multivariable | |
| --- | --- | --- | --- | --- |
|  | HR (95% CI) | *P* | HR (95% CI) | *P* |
| Age (continuous) | 0.99 (0.96 to 1.01) per score | 0.29 |  |  |
| Sex (female vs. male) | 1.54 (0.85 to 2.80) | 0.15 |  |  |
| ECOG PS (≥ 2 vs. ≤ 1) | 3.00 (0.71 to 12.76) | 0.14 |  |  |
| Primary site (upper urinary tract or both vs. bladder only) | 1.04 (0.59 to 1.84) | 0.89 |  |  |
| Resection of primary site (yes vs. no) | 0.79 (0.39 to 1.59) | 0.51 |  |  |
| Cockcroft-Gault creatinine clearance (continuous) | 1.00 (0.98 to 1.01) per score | 0.79 |  |  |
| Prior perioperative chemotherapy (yes vs. no) | 0.80 (0.34 to 1.88) | 0.60 |  |  |
| Cycles of dd-MVAC (continuous) | 0.83 (0.72 to 0.96) per score | 0.012 | 0.91 (0.78 to 1.09) per score | 0.30 |
| Response to dd-MVAC (≥ SD vs. PD) | 0.072 (0.033 to 0.16) | < 0.001 | 0.083 (0.031 to 0.22) | < 0.001 |
| Lymph node metastasis (yes vs. no) | 1.15 (0.64 to 2.06) | 0.65 |  |  |
| Lung metastasis (yes vs. no) | 1.65 (0.92 to 2.94) | 0.093 |  |  |
| Bone metastasis (yes vs. no) | 2.43 (1.28 to 4.64) | 0.007 | 1.03 (0.47 to 2.29) | 0.94 |
| Liver metastasis (yes vs. no) | 3.35 (1.66 to 6.77) | < 0.001 | 3.58 (1.73 to 7.42) | < 0.001 |

CI, confidence interval; dd-MVAC, dose-dense methotrexate, vinblastine, doxorubicin, and cisplatin; ECOG PS, Eastern Cooperative Oncology Group performance status; HR, hazard ratio; PD, progressive disease; PFS, progression-free survival; SD, stable disease
